# Life history and assessment of grapevine phylloxera leaf galling incidence on *Vitis* species in Uruguay

**DOI:** 10.1186/2193-1801-2-181

**Published:** 2013-04-23

**Authors:** María Valeria Vidart, María Valentina Mujica, Leticia Bao, Felicia Duarte, Carlos María Bentancourt, Jorge Franco, Iris Beatriz Scatoni

**Affiliations:** 1 Department of Plant Protection, Faculty of Agronomy, University of the Republic, Ave. E. Garzón 780, Montevideo, 12900 Uruguay; 2 Department of Biometry, Statistics and Computation, Faculty of Agronomy, University of the Republic, Ave. E. Garzón 780, Montevideo, 12900 Uruguay

**Keywords:** *Daktulosphaira vitifoliae*, Hemiptera: Phylloxeridae, Seasonal development, Foliage infestation

## Abstract

Grapevine phylloxera, *Daktulosphaira vitifoliae* (Fitch) (Hemiptera: Phylloxeridae) is a worldwide pest of *Vitis* species. It has forms that feed on leaves and roots. Root forms predominate on *Vitis vinifera* (L.) cultivars, while leaf forms predominate on *Vitis* species from its native American range. Recently, high densities of *D. vitifoliae* infestations in leaves of *V. vinifera* in Brazil, Peru, and Uruguay have been reported. The aims of this study were to determine the seasonal development of grape phylloxera, quantify infestation levels on *V. vinifera* leaves, and compare them with infestation levels on leaves of a rootstock of American origin. Studies were conducted in two vineyards in Uruguay from 2004–2007. Terminal shoots of 3309 C and Cabernet Sauvignon, Chardonnay, Tannat, Viognier, grafted onto resistant rootstock, were sampled weekly and leaves examined for gall presence and insect life stage. First galls were detected in early October; eggs began to appear within two weeks. Two oviposition peaks occurred by the end of December, and they coincided with bursts of shoot growth. On 3309C rootstock, oviposition peaks were more frequent than on the European cultivars. Based on thermal accumulation, *D. vitifoliae* could complete eight generations a year in Uruguay. Rootstock 3309C suffered the greatest damage but in some cases was similar to the European cultivars. Damage to Chardonnay, Cabernet Sauvignon and Viognier were also high. There were no galls on Tannat. The 2005–2006 season was characterized by low infestation rates caused by a prolonged drought that affected vegetative growth. There were also differences between vineyards, where the vigorous plants suffering more damage. Leaf galling phylloxera incidence and damage were mainly associated to the cultivar but plant vigor and environmental factors also contributed to increase the incidence.

## Introduction

Grapevine phylloxera, *Daktulosphaira vitifoliae* (Fitch) (Hemiptera: Phylloxeridae), is an insect pest of *Vitis* species. It has forms that feed on leaves (gallicolae) and roots (radicicolae). On *Vitis* species native to North America, *D. vitifoliae* induces galls on leaves and feeds on roots without evident injury. The real impact of grape phylloxera occurred when it was accidentally introduced in Europe and the root forms devastated European grapevine *Vitis vinifera* L., first in France, then across the continent and eventually the world (Grannet *et al.*^2001^).

Consequently, intense interest in this insect emerged. Most modern rootstocks used to control this pest were identified as a result of 19th century research. The resistance of these rootstocks has been extraordinarily persistent, so interest in the bio-ecology of the insect and its damage declined over time. New research has been conducted only intermittently, each time a new viticulture area was colonized or a widely-used rootstock has failed to control *D. vitifoliae* (Grannet *et al.*^2001^).

The first vines planted in Uruguay were imported by Spanish colonists in the late 18th century; however commercial viticulture has its origins between 1860 and 1870 when two commercially important vineyards were established, one in the north and one in the south of the country. Grape phylloxera was detected on these vineyards 20 years later, and as in Europe, Uruguayan viticulture was restored through the use of resistant rootstocks, a practice that persists today (Echeverría 2005).

Currently, Uruguay has a vineyard area of approximately 8,000 hectares (DIEA–MGAP 2011) planted mostly with high-quality stock that has been selected and sanitized, thanks to a Vineyard Recovery Plan between 1997 and 2003 (Macagno 2006). Recently, new plantations have suffered *D. vitifoliae* infestations on *V. vinifera* leaves in very high densities and at various locations. Similar situations have been observed in Italy, France, the United States (New York), Peru (Grannet *et al.*^2001^), Brazil (Botton and Walker 2009), Hungary (Molnár *et al.*^2009^) and Austria (Könnecke *et al.*^2011^).

This increased occurrence worldwide of leaf-galling grape phylloxera on *V. vinifera* leaves was unexpected and could have several causes including emergence of new endemic biotypes, breakdown in resistance of novel selected cultivars, or accidental introduction of exotic biotypes on planting material (Grannet *et al.*^2001^).

Uruguayan vineyards are characterized by the lack of insects or mites with systematic effects (Bentancourt and Scatoni 2010). The use of insecticides to manage this crop is an exception, so any pest species that exceeds damage thresholds and requires control measures will alter the population balance of beneficial organisms.

Briozzo and Carbonell (1980 and Scatoni *et al.*(1981)) studied the biology of grape phylloxera in the laboratory and field in Uruguay. These investigations have been recently resumed because of the resurgence of severe phylloxera infestation in the foliage of some *V. vinifera*. The aims of this study were to determine the seasonal development of *D. vitifoliae* to foliage infestation, examine the sensitivity of new *V. vinifera* clones to the leaf-galling grape phylloxera and compare them with infestation levels to leaves of a rootstock of American origin.

## Materials and methods

The study was carried out in two commercial vineyards, 10 km apart, in Canelones Department, Uruguay, over three consecutive years (2004–2007). One site was located in Juanicó (34°58′S, 56°25′W; 200 hectares) and the other in Progreso (34°68′S, 56°21′W; 50 hectares). The vineyard located in Juanicó was planted on soil with over 150 years of grapevine history, with average organic matter content of 1.9%, while the vineyard in Progreso was installed on uncultivated soil, without previous history of grapevine and organic matter content ranged between 3.6 and 4.6% depending on the block. In each location, the *V. vinifera* cultivars Cabernet Sauvignon, Viognier, Chardonnay and Tannat, grafted on SO4 rootstock (*V. berlandieri* x *V. riparia*), planted in adjacent blocks were evaluated. In Juanicó, assessments were also performed on a block of American rootstock hybrid 3309 C (*V. riparia* x *V. rupestris*).This rootstock and the Chardonnay cultivar in Juanicó were not assessed in the 2006–2007 growing season because they were removed in the winter. The characteristics of the vineyards are presented in Table 1. Insecticides were not applied in either vineyard during the study period, from 2004–2007.

**Table 1:** **Characteristics of the vineyards located at Progreso and Juanicó, Uruguay**

Location	Cultivar	Year planted
Progreso	Cabernet Sauvignon	1996
	Chardonnay	1999
	Tannat	1999
	Viognier	1999
Juanicó	Cabernet Sauvignon	1994
	Chardonnay	1986
	Tannat	1994
	Viognier	1992
	3309 C	Dateless

Every week, from October to March, 10 terminal shoots (15 cm long) were randomly sampled with an average of 6–7 unfolded leaves from each cultivar. In the laboratory, the proportion of leaves with phylloxera galls (a measure of damage incidence) and the number of galls per infested leaf (a measure of severity) were recorded. To evaluate grape phylloxera seasonal development, 30 galls per week and cultivar were randomly taken from the shoot samples. These galls were dissected under stereo-microscope and the presence and insect life stages inside were recorded. Some samples had no galls or too few to evaluate developmental stages.

To predict pest population development, degree day (DD) accumulation from October 1^st^ (biofix) was estimated using 6.4°C as a lower threshold temperature (LTT) and 303 DD as a thermal constant (K) to complete a generation (Johnson *et al.*^2010^, Sleezer et al. 2011). Degree days were estimated using the Baskerville and Emin (1969) method based on maximum and minimum air temperatures. Daily maximum and minimum temperatures and precipitation were taken from the Experimental Station of the National Agricultural Research Institute Las Brujas from 2004–2007. This station is located 10 and 12 km, respectively, from the Progreso and Juanicó vineyards.

Statistical analyses were done in the framework of generalized linear models (McCullagh and Nelder 1999) assuming a binomial distribution and a logit link function for percentage of leaves with galls and a Poisson distribution with log link function for the number of galls per infested leaf. Finally, to compare incidence and severity of the damage on grape cultivars within each locality in different years, and also to contrast the mean of these variables between locations and years, for cultivars that were present at the two locations in the three years, we applied χ2 tests. Analyses were done using the GENMOD procedure in SAS v. 9.2 (SAS Institute 2009).

## Results and discussion

### Seasonal development of foliage infestation

**Figure 1:**
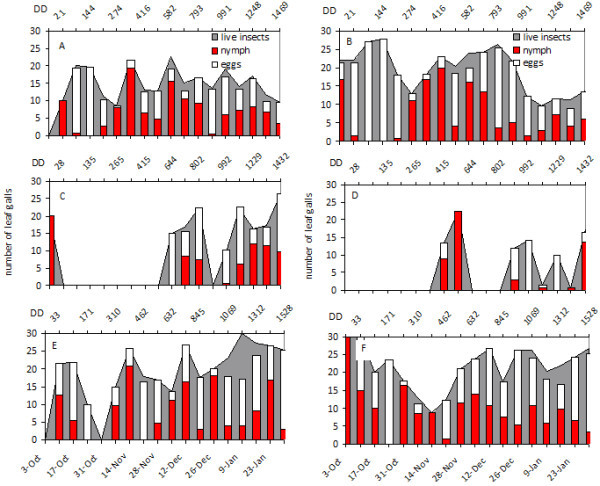
**Grapevine phylloxera leaf-gall infestation levels in Cabernet Sauvignon.** (**A C E**) Juanicó, (**B D F**) Progreso, (**A B**) 2004-2005, (**C D**) 2005-2006, (**E F**) 2006-2007. Degree days are shown in the secondary X axis. Numbers of galls containing live insects (grey background), eggs (white bars), and nymphs (red bars) are indicated.

**Figure 2:**
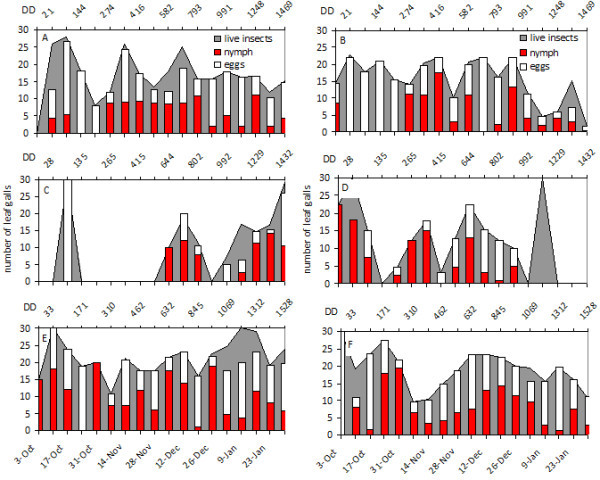
**Grapevine phylloxera leaf-gall infestation levels in Viognier.** (**A C E**) Juanicó, (**B D F**) Progreso, (**A B**) 2004-2005, (**C D**) 2005-2006, (**E F**) 2006-2007. Degree days are shown in the secondary X axis. Numbers of galls containing live insects (grey background), eggs (white bars), and nymphs (red bars) are indicated.

**Figure 3:**
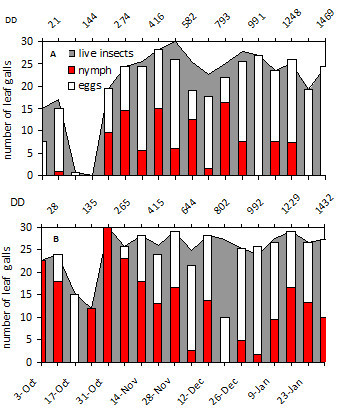
**Grapevine phylloxera leaf-gall infestation levels in rootstock 3309 C in Juanicó.** (**A**) 2004-2005, (**B**) 2005-2006. Degree days are shown in the secondary X axis. Numbers of galls containing live insects (grey background), eggs (white bars), and nymphs (red bars) are indicated.

Representative graphs are shown for the proportion of leaf galls containing live insects, eggs and four nymphal instars for Cabernet Sauvignon and Viognier in Progreso and Juanicó (Figures 1 and 2, respectively) and 3309 C in Juanicó (Figure 3) for the first two seasons of study, because they were removed after the second season.

In the 2004–2005 and 2006–2007 growing seasons, the first galls with nymphs were found in early October (Figures 1 and 2); these nymphs probably colonized the vegetation up from the roots and rhytidome, considered the main overwintering site. Two weeks later, female oviposition peaked, and eggs began to hatch after approximately 7 days. Through December there were two peaks of oviposition, in general, one in mid-November and the other in December (Figures 1 and 2), coinciding with the greatest abundance of shoots propitious for insect development. This behavior was consistent in both localities and in all European cultivars studied, except Tannat. We found no galls on leaves of Tannat. At least three other generations, which were not clearly defined, could occur from January to March (unpublished data).

On the rootstock 3309 C, oviposition peaks were observed more frequently than in the other cultivars (Figure 3); this stock may have a more suitable and abundant food throughout the growing season. 3309 C had the lowest proportion of empty or malformed galls, followed by Cabernet Sauvignon and Viognier. However, several galls were empty in Chardonnay, which would indicate that the insect had difficulty establishing on leaves of this cultivar (unpublished data).

A different pattern was observed in the 2005–2006 growing season; Cabernet Sauvignon in both localities and Viognier in Juanicó experienced a 1.5-month delay in the first appearance of leaf galls that could not be explained by a lower accumulation of DD (Figures 1 and 2). Accumulated DD in October and November of all three years was very similar, so the predicted beginnings of the second and third generation based on K were also similar (Table 2). The population fluctuations in each year did not vary substantially among cultivars or between locations.

**Table 2:** **Predicted start dates of the second and third generations of leaf-form nymphs of*Daktulosphaira vitifoliae*in Canelones Department, Uruguay**

Year	Biofix	Start date 2^nd^generation	Start date 3^rd^generation
		(K^1^ = 303 DD, LTT^2^ = 6.4°C)	(K = 606 DD, LTT = 6.4°C)
**2004**	1-Oct	2-Nov	30-Nov
**2005**	1-Oct	3-Nov	26-Nov
**2006**	1-Oct	30-Oct	25-Nov

Note: Dates were estimated from accumulated degree days since October 1^st^ of each year.

^**1**^ Thermal constant and ^**2**^Lower threshold temperature determined by Johnson *et al.*^2010^.

Development of leaf galling *D. vitifoliae* and associated leaf-galls depends on the quality of substrate and the environmental temperature and humidity (Flaherty *et al.*^1992^). The seasonal development of leaf galling phylloxera under the conditions of this study would seem more strongly affected by the vegetative growth of the vines than with thermal accumulation over the threshold of insect development, although in the 2004–2005 and 2006–2007 seasons development correlated to heat accumulation (Table 2, Figures 1 and 2). Grape phylloxera populations usually increased from bud break until shoot production stabilized in midsummer. Sprouting slows in mid-December which, combined with canopy management and green-stage pruning, makes it difficult to locate new leaf galls by removing vegetative tips and lateral shoots in *V. vinifera* cultivars. Although sampling continued into March, those data were not graphed because the galls remaining on the plants were restricted to mature leaves and generally contained only dead insects or remnants of hatched eggs rather than developing insects. On 3309 C where vegetative growth was continuous, insect generations occurred continuously and with substantial overlap of developmental stages (Figure 3). Based on thermal accumulation, *D. vitifoliae* could complete eight generations a year in Uruguay from October to March, but we could not determine this, due to the large overlap of development stages at the end of the season.

### Assessment of damage to foliage

**Figure 4:**
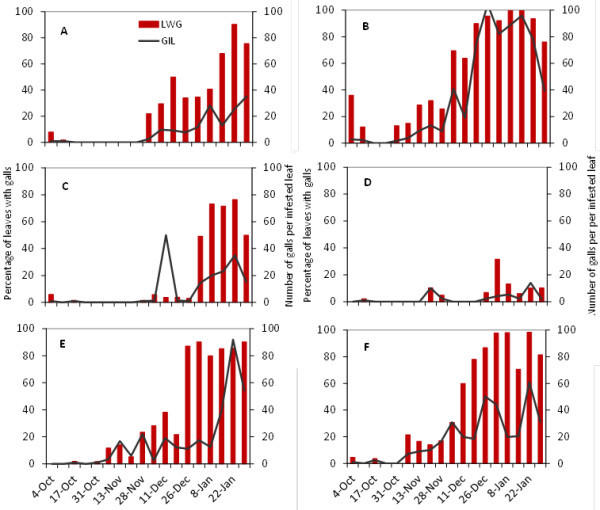
**Percentage of leaves with galls (LWG) and number of galls per infested leaf (GIL) in Cabernet Sauvignon.** (**A C E**) Juanicó, (**B D F**) Progreso, (**A B**) 2004-2005, (**C D**) 2005-2006, (**E F**) 2006-2007.

**Figure 5:**
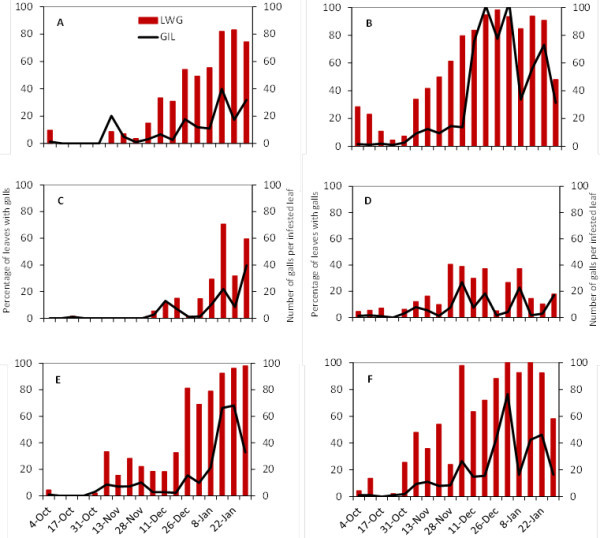
**Percentage of leaves with galls (LWG) and number of galls per infested leaf (GIL) in Viognier.** (**A C E**) Juanicó, (**B D F**) Progreso, (**A B**) 2004-2005, (**C D**) 2005-2006, (**E F**) 2006-2007.

**Figure 6:**
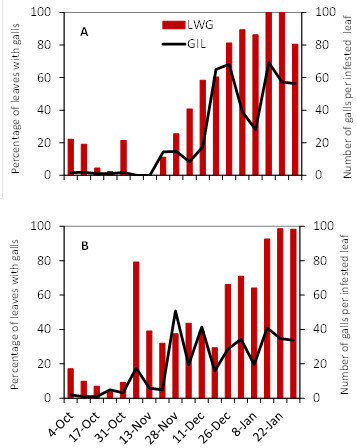
**Percentage of leaves with galls (LWG) and number of galls per infested leaf (GIL) in the rootstock 3309 C grown in Juanicó.** (**A**) 2004-2005, (**B**) 2005-2006.

Figures 4 and 5 show the incidence and severity of grape phylloxera leaf galls on Cabernet Sauvignon and Viognier during the three years of the study. In general, pest incidence was higher in the Progreso vineyard and moderate in Juanicó. Seasonal differences were evident. Clearly, the leaf galling incidence in the 2005–2006 growing season was different from those in other years. In the 2004–2005 season in Progreso, 100% of leaves from terminal shoots had galls, with an average of 100 galls per leaf, by late December, which did not happen in 2005–2006 on any of the European cultivars. In that season, only the rootstock 3309 C reached 100% infestation and not until January (Figure 6).

Table 3 summarizes the statistical analyses of the percentage of leaves with galls and the number of galls per infested leaf (October–January), for each cultivar, season, and locality. In Juanicó, rootstock 3309 C showed the highest damage, although in some cases the incidence was similar to that observed in the cultivars Cabernet Sauvignon and Viognier. In this locality, Cabernet Sauvignon and Viognier showed no differences between them comparing the percentage of leaves with galls, but in the 2005-2006 season differed in the severity of damage. Chardonnay was the least attacked cultivar in Juanicó, the lower infestation rates might be explained by their lack of vigor, as they were the oldest vines in this study (Strapazzon and Girolami 1983). Chardonnay, Cabernet Sauvignon and Viognier in Progreso showed similar damage, with narrower differences depending on the season. Tannat was not attacked by *D. vitifoliae* in either of the vineyards in the three years of the study, even when surrounded by infested vineyards, so it was not included in the statistical analysis. Its leaves are probably unfavorable for the insect, but unknown factors could also cause this resistance. The analysis indicated that the average incidence in 2005–2006 was lower in Progreso, but no clear difference among years was evident in Juanicó. However, when comparing only the European cultivars Cabernet Sauvignon and Viognier at both sites and in the three years, on average the 2005-2006 season was different from the others and the incidence and severity of the damage was higher in Progreso, without statistical differences among cultivars (Table 4).

**Table 3:** **Percentage of leaves with galls (LWG) and galls per infested leaf (GIL), for five grape cultivars, in Juanicó and Progreso, Uruguay**

Growing season	Cultivar	Juanicó				Progreso			
		Mean LWG		Mean GIL		MeanLWG		Mean GIL	
2004–2005	3309C	58.10	a*	45.36	a				
	Chardonnay	1.20	c	4.33	d	51.79	b	21.39	d
	C. Sauvignon	31.81	b	16.16	d	67.70	ab	70.25	a
	Viognier	37.05	ab	17.80	d	70.40	ab	55.94	b
	Tannat**	0.00				0.00			
2005–2006	3309C	55.74	a	28.63	b				
	Chardonnay	2.86	c	16.31	d	8.80	cd	14.36	e
	C. Sauvignon	23.00	b	23.05	c	6.68	d	5.79	e
	Viognier	14.51	bc	13.55	d	22.09	c	12.25	e
	Tannat	0.00				0.00			
2006–2007	3309C								
	Chardonnay					81.18	a	30.18	c
	C. Sauvignon	43.99	a	30.63	b	54.05	b	33.08	c
	Viognier	46.11	a	29.20	b	70.72	ab	32.12	c
	Tannat	0.00				0.00			

* Numbers in a column followed by the same letter are not statistically different (*P* > 0.05) according to the χ^2^ test when comparing the cultivars in each locality.

** Tannat was not included in the statistical analysis.

**Table 4:** **Percentage of leaves with galls (LWG) and galls per infested leaf (GIL), for two grape cultivars and three years of study, in Juanicó and Progreso, Uruguay**

**Growing season**	**Mean LWG**		**Mean GIL**	
2004-2005	51.13	a*	47.15	a
2005-2006	16.66	b	15.84	b
2006-2007	53.66	a	31.41	a
**Locality**	**Mean LWG**		**Mean GIL**	
Juanicó	32.10	b	22.84	b
Progreso	47.59	a	45.76	a
**Cultivar**	**Mean LWG**		**Mean GIL**	
Cabernet Sauvignon	36.79	a	39.37	a
Viognier	42.79	a	33.52	a

Note: Comparing the average damage by growing season, locality and cultivar.

* Numbers in a column followed by the same letter are not statistically different (*P* > 0.05) according to the χ^2^ test when comparing the growing seasons, localities and cultivars.

We inferred that the lower incidence of infestation in 2005–2006 might be due to climatic factors acting directly and indirectly on the insect, mainly by affecting the quality of its food. The limited rainfall from August to December 2005 affected vine sprouting and consequently the ability of *D. vitifoliae* to establish galls and reproduce. October is the key month for grape phylloxera establishment, because that is when juveniles ascend from the roots to the foliage; in 2005, October was characterized by both low rainfall and above-normal temperatures (Table 5). According to Korosi *et al.* (2012) high temperatures combined with low relative humidity cause mortality of *D. vitifoliae* dispersive stages in less than two hours.

**Table 5:** **Monthly accumulated precipitations (mm) recorded during this study and of a historical series in Canelones Department, Uruguay**

Month	Precipitation (mm)			
	2004–2005	2005–2006	2006–2007	1961-1990^1^
March	57	70	163	105
April	212	184	30	86
May	33	93	19	89
June	67	157	245	83
July	51	91	83	86
August	58	46	35	88
September	46	76	41	94
October	156	64	129	109
November	110	38	84	89
December	62	24	112	84
January	199	233	36	87
February	117	50	164	101

^1^Dirección Nacional de Meteorología, Uruguay, http://http//www.meteorologia.gub.uy/index.php/estadísticas-climaticas.

Leaf galling phylloxera incidence and damage were mainly associated to the cultivar (Table 3). The difference between vineyards might be due to plant vigor; overall, the grapevines at the Progreso site were younger (Table 1) and growing in a more fertile soil without previous grapevine history that made the grapevines unevenly balanced with exuberant sprouting. This finding coincides with the observations of Strapazzon and Girolami (1983 for Italy and Molnár *et al.* ()2009) for Hungary, who reported leaf galls on vigorous plants of *V. vinifera*. However, we do not reject the hypothesis that different biotypes exist in both vineyards, which is now being studied (unpublished data).

For gall-forming insects to be successful, they must be present when the target organ is susceptible. Therefore, gall-formers preferably use actively-growing plant tissues (Bauerle *et al.*^2007^). Thus, a more vigorously-growing plant would have more new shoots available to herbivores than a stressed plant (Grannet *et al.*^2001^), which could explain our results. Abundant rains in the spring 2004 and 2006 favored vegetative growth and consequently the leaf galling *D. vitifoliae* incidence in those seasons. Most damage in Progreso was also due to a higher vegetative growth associated with greater soil fertility and younger plants.

Grape phylloxera uses many *Vitis* species as hosts. The complex interactions among grape cultivar and physiology, environmental conditions, and the phylloxera biotype and its physiology that affect gall formation have a mechanistic basis that has not yet been explained. Because gall formation is a prerequisite for phylloxera survival and growth and the consequent vine damage, understanding these mechanisms has a great practical significance (Grannet *et al.*^2001^).

Our results, observations and experiences confirm the necessity for an in-depth investigation of grape phylloxera leaf galling, especially on the factors that could be leading to the different susceptibility among cultivars of *V. vinifera*. It is clear that environmental factors (climate, soil) and grape phylloxera biotypes may be contributing. However, the cultivar Tannat in both vineyards was growing under the same environmental conditions, came from the same nursery, had the same age, was surrounded by infested cultivars and did not presented leaf galls throughout this study. Tannat is the predominant cultivar in Uruguay, their sources of resistance to leaf galling *D. vitifoliae* should be clarified, and maybe they could be useful in future plant breeding programs.
